# Original Contributions to the Chemical Composition, Microbicidal, Virulence-Arresting and Antibiotic-Enhancing Activity of Essential Oils from Four Coniferous Species

**DOI:** 10.3390/ph14111159

**Published:** 2021-11-13

**Authors:** Diana-Carolina Visan, Eliza Oprea, Valeria Radulescu, Ion Voiculescu, Iovu-Adrian Biris, Ani Ioana Cotar, Crina Saviuc, Mariana Carmen Chifiriuc, Ioana Cristina Marinas

**Affiliations:** 1 Department of Organic Chemistry, Faculty of Pharmacy, University of Medicine and Pharmacy “Carol Davila”, 6 Traian Vuia Street, 020956 Bucharest, Romania; diana.ilies@umfcd.ro (D.-C.V.); valeria_radulescu@yahoo.com (V.R.); 2 Department of Organic Chemistry, Biochemistry and Catalysis, Faculty of Chemistry, University of Bucharest, 4-12 Regina Elisabeta, 030018 Bucharest, Romania; 3 “Marin Drăcea” National Institute for Forestry Research and Development, 128 Eroilor, 077190 Voluntari, Romania; ion.voiculescu@yahoo.com (I.V.); iovu.biris@gmail.com (I.-A.B.); 4 Faculty of Agriculture, University of Agronomic Sciences and Veterinary Medicine of Bucharest, 59 Mărăşti, 011464 Bucharest, Romania; 5 Cantacuzino National Medico-Military Institute for Research and Development, 103 Splaiul Independenței, 050096 Bucharest, Romania; aniioana@gmail.com; 6 Research Institute of the University of Bucharest—ICUB, 91-95 Spl. Independentei, 050657 Bucharest, Romania; crina.saviuc@yahoo.com (C.S.); carmen.chifiriuc@unibuc.ro (M.C.C.); ioana.cristina.marinas@gmail.com (I.C.M.); 7 Microbiology Department, Faculty of Biology, University of Bucharest, 1-3 Portocalilor Way, 060101 Bucharest, Romania; 8 Academy of Romanian Scientists, 3 Ilfov Street, 50044 Bucharest, Romania

**Keywords:** essential oil, antimicrobial activity, *Picea abies*, *Larix decidua*, *Pseudotsuga menziesii*, *Pinus nigra*, quorum sensing

## Abstract

This study aimed to establish the essential oil (EO) composition from young shoots of *Picea abies*, *Larix decidua*, *Pseudotsuga menziesii*, and *Pinus nigra* harvested from Romania and evaluate their antimicrobial and anti-virulence activity, as well as potential synergies with currently used antibiotics. The samples’ EO average content varied between 0.62% and 1.02% (mL/100 g plant). The mono- and sesquiterpene hydrocarbons were dominant in the composition of the studied EOs. The antimicrobial activity revealed that the minimum inhibitory concentration (MIC) values for the tested EOs and some pure compounds known for their antimicrobial activity ranged from 6.25 to 100 µL/mL. The most intensive antimicrobial effect was obtained for the *Pinus nigra* EO, which exhibited the best synergistic effect with some antibiotics against *Staphylococcus aureus* strains (i.e., oxacillin, tetracycline, erythromycin and gentamycin). The subinhibitory concentrations (sMIC) of the coniferous EOs inhibited the expression of soluble virulence factors (DN-ase, lipase, lecithinase, hemolysins, caseinase and siderophore-like), their efficiency being similar to that of the tested pure compounds, and inhibited the rhl gene expression in *Pseudomonas aeruginosa*, suggesting their virulence-arresting drug potential.

## 1. Introduction

Plant essential oils (EOs) are complex mixtures of volatile natural compounds. EOs are formed in aromatic plants as secondary metabolites (terpenes, terpenoids, phenylpropenes and “others”) [1], which play an important role in plant defense [2] and have been used since ancient times as natural remedies for fighting infectious diseases caused by different microbial and viral pathogens [3,4,5]. They are relatively easy to obtain, have low mammalian toxicity, and degrade quickly in water and soil, making them relatively environmentally friendly [1].

It has been shown that plant extracts have pronounced antimicrobial activities, even when used in subinhibitory concentrations, which do not interfere with bacteria growth but only with their behavior [6], leading to a reduced risk of developing resistance to that compound and to a lower risk of dysbiosis [7]. Moreover, these low concentrations will have minimal or no effect against host cells. Taking into account these aspects, the most recent anti-infective approaches, also called anti-pathogenic or anti-virulence strategies, propose targeting virulence factors expression and biofilm development rather than inhibition of microbial growth or killing the pathogens [6,8]. Despite their superior resistance to antibiotics, biofilm-embedded bacteria seem to be more susceptible than their planktonic counterparts to some EOs, probably because (i) the extracellular matrix of the biofilm adsorbs the active phytocomponents and increases their local concentration; and (ii) the cellular envelope (capsule, cellular wall and membrane) in biofilm cells is different from that of free cells due to differential gene expression in the two growth states and more susceptible to EOs [9].

For this study we have chosen to evaluate the EOs of four coniferous species, being known that coniferous forests are a renewable source of EOs that are distributed in various organs of these plants: needle/leaves, roots, cones/seeds, wood/stem/twigs, bark and berries [8].

The EOs main compounds of coniferous species are monoterpenes, monoterpenoides, sesquiterpenes, sesquiterpenoides and diterpenes [10,11]; however, the chemical composition of EOs could be variable, depending on the anatomical part of the tree, the genetic factors [12], the health condition of plant and also on the geographic and environmental conditions: soil and water composition, humidity and air pollution [13,14,15]. Research conducted in recent decades have highlighted the antibacterial [16,17], antifungal [17,18,19,20] and antioxidant [21,22] properties of EOs isolated from different coniferous species [15,19,22,23,24,25,26,27,28,29,30] but there is scarce information concerning the biologically active principles isolated from populations of coniferous species on the Romanian territory. This type of research is very important if we consider that Romania is the first place in Europe regarding the rates of antimicrobial resistance [11,31]. We have previously shown that the *Abies alba* EO inhibits agrI gene expression in *Staphylococcus aureus*, suggesting an inhibitory effect on the quorum sensing (QS) genes expression and indirectly on the strain virulence, and therefore their anti-pathogenic potential [32].

The objectives of this study were to investigate the composition of the EOs from young shoots of four coniferous species and to evaluate their antimicrobial activity. The biological material consisted of three Eurasian species native to the Carpathian area—spruce (*Picea abies*), larch (*Larix decidua*) and black pine (*Pinus nigra*)—and a species of North American origin—the Douglas fir (*Pseudotsuga menziesii*)—naturalized and often used in forest plantations in Europe.

## 2. Results and Discussion

### 2.1. Essential Oil Content and Composition

**Figure 1:**
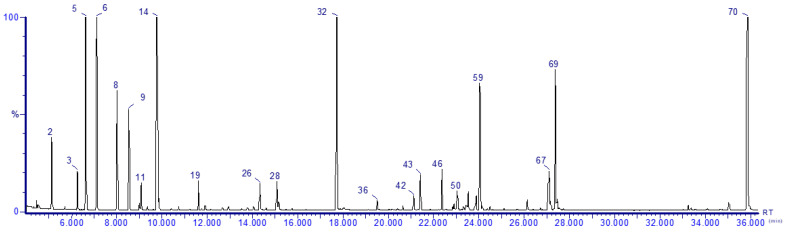
Chromatogram of the *P. abies* essential oil.

The average EO content of the *P. abies*, *L. decidua*, *P. menziesii* and *P. nigra* samples (five determinations for each sample) was 1.02 ± 0.19, 0.62 ± 0.13, 0.87 ± 0.04 and 0.82 ± 0.14% (mL essential oil/100 g dried plant), respectively. Figure 1 shows the chromatogram of the *P. abies* EO and in Table 1 are listed the identified compounds for all four EO samples. The other chromatograms of the EOs are included in Supplementary Materials (Figures S1–S3). The numbers from the peak of the compounds on the four chromatograms correspond to the numbers listed in Table 1.

The main compound classes from the studied EOs were mono- and sesquiterpene hydrocarbons, representing 69.20% (*P. abies*), 84.53% (*L. decidua*), 93.72% (*P. menziesii*) and 94.39% (*P. nigra*) from each EO.

Camphene, α-pinene, β-pinene and limonene are prevalent among the monoterpene hydrocarbons. The EO compositions isolated from *P. abies* [11], *L. decidua* and *P. menziesii* [34] are similar to the previously published papers. For the EO extracted from *P. abies*, the content in compounds with oxygen is significantly higher than in other oils, i.e., 11.08% for bornyl acetate, 9.40% for manool, 3.87% for α-cadinol, and 2.15% for α-muurolol. Germacrene D is the major compound (19.80%) in the EO of *Larix decidua*, while pinene (α and β) is one of the most important components of the EO extracted from *P. abies*, these data being in accordance with those reported by Mofikoya (2020) [35].

### 2.2. Antimicrobial Activity

#### 2.2.1. Qualitative and Quantitative Analysis

The qualitative screening of the EOs and the pure compounds revealed the occurrence of a growth inhibition zone in the area where the EOs: DMSO stock solution was spotted. We started by assessing the efficiency of the tested EOs against a larger batch of microbial strains, but only those strains for which a growth inhibition zone was observed were further tested by quantitative assay. Thus, in Table 2 are presented only the strains that proved to be sensitive to the EOs and pure compounds when tested by the qualitative assay.

The quantitative assay revealed that the MIC values for the tested EOs as well as for some pure compounds known for their antimicrobial activity ranged from 6.25 to 50 µL/mL, the most intensive effect being obtained for *P. nigra*, exhibiting the lowest MIC values against all tested strains (Table 2).

Concerning the antimicrobial activity of the pure compounds, the most active proved to be α-pinene, *S. aureus* strains being more susceptible than the Gram-negative ones. Phellandrene, borneol and camphor had the same effect as nerolidol. The EOs proved also to be more effective against *S. aureus* strains, as well as towards *Bacillus subtilis* and *Candida albicans* strains, as compared to the Gram-negative species. It is to be noticed that in many cases, the MIC values were lower for the EOs than those obtained for the pure compounds, demonstrating the synergistic effect of the active compounds found in the EOs. The antimicrobial activity of *P. abies* EO could mainly be due to bornyl acetate because this compound has shown a good activity on *S. aureus* (MIC 1.95 mg/mL), *P aeruginosa* (MIC 2.30 mg/mL) and *Escherichia coli* (MIC 4.88 mg/mL) [36]. Some studies showed that α-pinene and β-pinene are able to destroy the cellular integrity by inhibition of respiration and ion transport processes [37,38]. Helander et al. [39] showed that the low molecular mass lipophilic compounds are responsible for the toxicity of EO components on Gram-negative bacteria because these compounds are able to penetrate the bacterial membrane and may thus be able to influence the proliferation of certain pathogenic bacteria.

**Figure 2:**
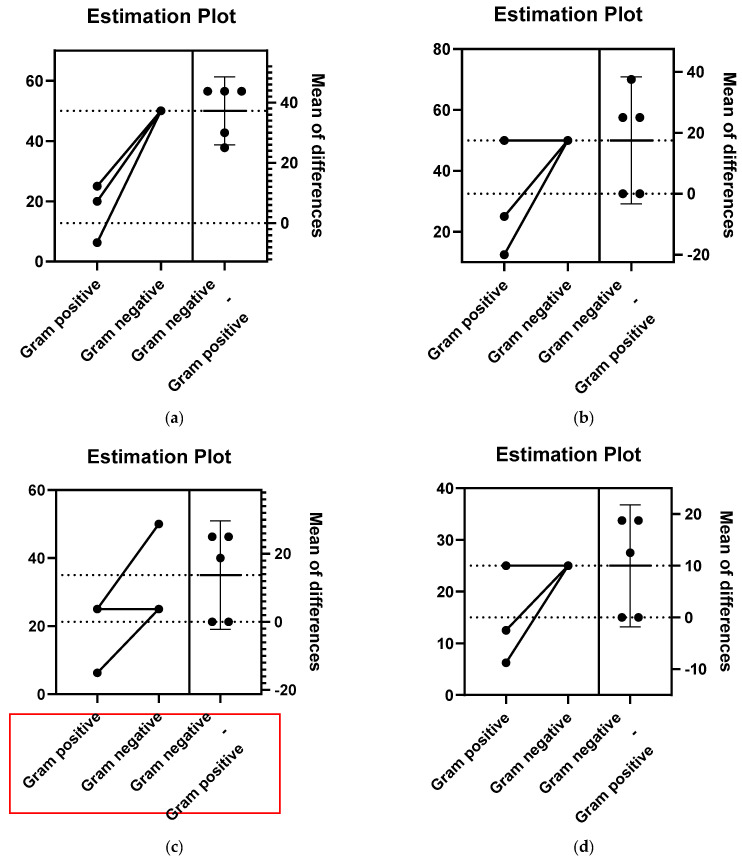
Estimation plots of the antibacterial activity of the *P. abies* (**a**), *L. decidua* (**b**), *P. menziesii* (**c**) and *P. nigra* (**d**) EOs. The Gram-negative–Gram-positive effect size is generated by the difference between means. The precision of the calculated effect size was at a 95% confidence interval.

By comparing the effect of the *P. abies* EO on Gram-positive and Gram-negative strains, a statistically significant activity was observed for Gram-positive bacteria (*p* < 0.001) (Figure 2a). For *L. decidua*, *P. menziesii* and *P. nigra*, the difference between the antimicrobial activity on Gram-positive and Gram-negative strains was not significant (*p* > 0.05), despite the fact that a better effect was observed on Gram-positive bacteria (Figure 2b–d).

According to Magwa et al. [40], *Sesuvium portulacastrum* exhibited an antibacterial activity against *S. aureus*, which may be due to the camphene found in its EO. This compound, identified in the analyzed conifer EOs, seems to be responsible for their antibacterial activity, especially on Gram-positive bacteria, because *P. abies* has the highest concentration of camphene and the best effect on *S. aureus* and *B. subtilis*. The presence of *trans*-caryophyllene and camphene, known to possess antifungal activity [40,41], in the studied EO composition explain the effect on the *Candida* strain. The *P. abies* EO had the biggest percent of these compounds (camphene: 10.7% and *trans*-caryophyllene: 1.17%) and the best antifungal activity, followed by *P. nigra* (with camphene: 1.24% and *trans*-caryophyllene: 1.99%).

#### 2.2.2. The Adherence Capacity to the Inert Substrate

In the natural environment, but also in the infected host, microorganisms usually produce extracellular capsular polymers, mostly polysaccharides, known as a capsule, slime, or glycocalyx, which, in the case of pathogenic strains, are an important virulence factor, being involved in adhesion and colonization of inert substrata, such as medical devices [42].

The adherence capacity to the inert substrate of the reference and clinical strains was inhibited by all the tested EOs and pure compounds at subinhibitory concentrations, respectively, MIC/2. The results are represented as the minimum biofilm eradication concentration (MBEC) values in Table 2. These results show the promising potential of these EOs to appropriately address the challenges of biofilm-associated infections diagnosis and treatment, often remaining unresolved with the present approaches [43,44].

#### 2.2.3. The Synergistic Activity with Antibiotics

The tested EOs potentiated the currently used antibiotics against *S. aureus* and *P. aeruginosa* strains, the most intensive effect being observed in case of *P. nigra*. The *S. aureus* 12 H strain, in the presence of EOs obtained from *P. abies* and *P. menziesii*, switched from resistant to susceptible to oxacillin and tetracycline, and in the presence of the *P. nigra* EO, to erythromycin, while *S. aureus* 35 PL became susceptible to gentamycin (Table 3). In case of the *P. aeruginosa* strains, the growth inhibition diameters for piperacillin, ticarcillin-clavulanic acid, imipenem, aztreonam, ceftazidime, ciprofloxacin, colistin and gentamycin were not modified by the EOs, probably due to the multi-drug resistance phenotype of these strains, which is often mediated by efflux pumps that are not substrate-specific, thus being able to provide cross-resistance to the EOs [45].

The synergic effects could be produced by *α*-pinene, according to Kovač, who reported that the MICs reduced from 32 to over 512-fold when (−)-*α*-pinene was applied in combination with erythromycin, ciprofloxacin or triclosan [46].

Other recent studies revealed that numerous plant-derived compounds and EOs, by interfering with adherence, biofilm formation and motility, are also affecting antibiotic susceptibility [47,48,49].

#### 2.2.4. The Influence of EOs on the QS Genes Expression

Taking into account the involvement of *P. aeruginosa* strains in the etiology of opportunistic and nosocomial infections, as well as their high resistance rates to the current antibiotics, a significant number of the respective strains isolated from clinical specimens have been tested in order to establish the modulatory effect of the EOs on the expression of QS genes. A relatively new strategy for combating bacterial infections and resistance to antibiotics is represented by QS inhibitors (QSI). Some EOs [50,51,52,53] or their components [32,54,55] were already reported to inhibit QS genes expression. *P. aeruginosa*, a critical opportunistic nosocomial pathogen, produces different virulence factors synthesized under the control of QS systems *las* and *rhl*. The first one consists of *LasI*, which modulates the synthesis of the autoinducer N-(3-oxododecanoyl) homoserine lactone, and a transcriptional activator (*lasR*). The second is composed of a putative transcriptional activator, *rhlR* and *rhlI*, which manage the synthesis of N-butyryl homoserine lactone. An interconnecting role between these two systems in the QS hierarchy of *P. aeruginosa* is held by the PQS signaling system (which produces 2-heptyl-3-hydroxy-4-quinolone) [56].

**Figure 3:**
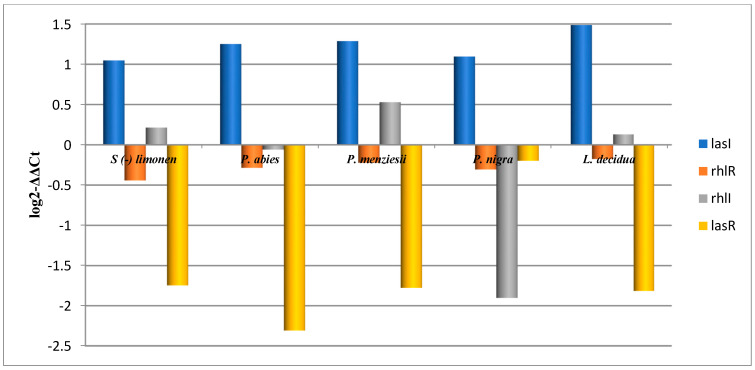
The QS genes expression levels in *P. aeruginosa* strains cultivated in the presence of limonene and EOs.

The rhl and lasR genes expression was significantly downregulated by the coniferous EOs, while lasI expression was upregulated (Figure 3).

According to Kostylev (2019), the RhlIR QS system requires induction by LasR, and as a consequence, a decreasing in the expression of QS-activated genes could be possible by lasR or lasI deletion in strain PAO1 (a laboratory model *P. aeruginosa* strain) [57].

As a result, these findings suggest that EOs interfere with the QS pathways in *P. aeruginosa* and could be a promising lead for the development of virulence-arresting drugs. These findings indicate that EOs may have an inhibitory effect on rhamnolipid production, which is regulated by the *P. aeruginosa* QS regulator *rhlR*, while an inhibitory effect on elastase and protease activities are regulated by the *rhlI-rhlR* system [58].

### 2.3. Influence of EO on the Expression of Soluble Enzymatic Virulence Factors

The tested strains have been previously analyzed for their virulence potential and selected as positive for producing the investigated virulence determinants, i.e., toxins forming pores in the membrane of eukaryotic cells (lecithinase, hemolysins and lipase), proteases (caseinase) and DN-ase [32].

Taking into account the rapid emergence of resistance to classical antimicrobial drugs, a new but expanding class, the so-called virulence-arresting drugs, targeting the inhibition of virulence factor production rather than kill pathogens, has emerged. These drugs can restore or augment the antibiotics’ effect in a pathogen-specific manner, thus decreasing the risk of resistance and side effects [59,60,61,62].

We have previously demonstrated the effects of some natural pure compounds or products (probiotic fractions, essential oils, bacteriophages, etc.) on the phenotypic or genotypic expression of virulence factors in opportunistic pathogens [32,63,64,65].

In the present study, the tested EOs inhibited the expression of the analyzed soluble virulence factors by different degrees, their efficiency being similar to that of the pure compounds. The most inhibited virulence factors in *S. aureus* were haemolysins, followed by siderophore-like compounds and lecithinase, while in *P. aeruginosa*, DN-ase, siderophore-like and haemolysins (Table 4).

## 3. Materials and Methods

### 3.1. Reagents and Solvents

SupraSolv dichloromethane was used for the gas chromatography, anhydrous Na_2_SO_4_ granulated for organic trace analysis, and the pure compounds (Ph. Eur.)—*α*-pinene, (+)-limonene, phellandrene, eucalyptol, borneol, camphor and nerolidol—were purchased from Merck, Darmstadt, Germany. The n-alkanes C_8_–C_24_ used for the determination of the Kovats retention indices were from Fluka, Switzerland.

### 3.2. Plant Material

The samples of young shoots with needles (around 1000 g) of Douglas fir (*P. menziesii*), European larch (*L. decidua* ssp. *Carpathica*), Norway spruce (*P. abies*) and black pine (*P. nigra* ssp. *nigra*) were collected from an intensive plantation located in the tree nursery of the “Marin Drăcea” National Institute for Forestry Research and Development (Voluntari, Romania). The plants grew in natural vegetation conditions, without the use of chemical fertilizers or pesticides to control weeds, diseases and pests. For each species, the samples were harvested from ten individual 6–8-year-old trees. The samples were dried, separated from branches and manually grounded.

### 3.3. Essential Oil Extraction

The needles (50 g) were hydro-distilled in a Clevenger-type apparatus for 4 h [66]. The EOs was dried over anhydrous Na_2_SO_4_, stored in a dark glass bottle and kept at 4 °C until analysis. The oil samples were diluted in dichloromethane (1/200) and 1 μL was injected for GC analysis.

### 3.4. Gas Chromatography–Mass Spectrometry

GC-MS analysis of the EOs was carried out using a Fisons Instruments GC 8000 with an electron impact quadrupole, MD 800 mass spectrometer detector.

The electron ionization energy was 70 eV. A fused silica column of 5% phenylpoly (dimethylsiloxane) (SLB–5 ms, 30 m × 0.32 mm i.d., film thickness = 0.25 μm) was employed. The operating conditions were as follows: a split-splitless injector (split ratio, 1:30) at 280 °C, ion-source temperature 200 °C and the interface temperature 280 °C; initial column temperature, 40 °C for 3 min, raised at 4 °C/min to 280 °C and finally held isothermally for 20 min; the carrier gas (helium) flow rate was 2 mL/min; and sample volume injected, 1 μL. Data acquisition was performed with MassLab 3.4 Software for the mass range 30–600 u with a scan speed of 1 scan/s. The identity of the EO components was established from their GC Kovats retention indices and from mass spectra by computer matching with a mass spectra library (NIST, Wiley and a personal library of 600 spectra). The Kovats retention indices were determined in relation to a homologous series of n-alkanes (C_8_–C_24_) and compared with those reported in the literature [67,68,69]. The components’ relative concentrations were calculated from the GC peaks without using correction factors.

### 3.5. Antimicrobial Activity

#### 3.5.1. Microbial Strains

The antimicrobial and anti-biofilm activity was tested on Gram-positive (*Staphylococcus aureus* ATCC 25923, *Bacillus subtilis* 6633, *Enterococcus faecalis* ATCC 29212) and Gram-negative (*Escherichia coli* ATCC 25922, *Pseudomonas aeruginosa* ATCC 27853) bacterial as well as fungal (*Candida albicans* ATCC 10231) reference strains, but also on *S. aureus* and *P. aeruginosa* strains isolated from hospitalized patients. *S. aureus* 19 F, *S. aureus* 8 V and *S. aureus* 35 PL were isolated from pharyngeal exudates, vaginal swabs and wound secretions, respectively; *S. aureus* 12 H and *P. aeruginosa* 1 H from blood cultures; and *P. aeruginosa* 61/2, *P. aeruginosa* 399 and *P. aeruginosa* 261/1 from urine cultures.

#### 3.5.2. Qualitative Assessment

The antimicrobial activity was determined by an adapted diffusion method. Briefly, the microbial inoculum with a density corresponding to 0.5/1 McFarland standard for bacterial/fungal strains was evenly swabbed on the agar surface in three directions, and thereafter, 10 μL of the stock solution of EO: DMSO was spotted on the seeded medium.

#### 3.5.3. Quantitative Analysis

Serial microdilution method in liquid medium using 96-well plates was performed, the intensity of bacterial growth being appreciated by the absorbance value read spectrophotometrically at 620 nm; the MIC (μL/mL) was determined as the last concentration at which no microbial growth was observed [70].

#### 3.5.4. The Microbial Adherence Capacity to the Inert Substratum

The slime test was used to highlight the EOs influence on the microbial adherence capacity to the inert substratum represented by the polymeric material of the 96 multi-well plates. Following the quantitative analysis of the antimicrobial effect, the adhered biomass was fixed with methanol, stained with violet crystal, resuspended in 33% acetic acid solution and assessed spectrophotometrically at 490 nm [71].

#### 3.5.5. The Influence of EOs on the QS Genes Expression

The effects of the EOs and limonene (EO or limonene: DMSO, 1:1, *v*/*v*) on QS gene expression in *P. aeruginosa* were investigated by real-time reverse transcriptase quantitative PCR (RT–qPCR), using a commercial kit (GeneJet RNA Purification Kit Fermentas), following the manufacturer’s indications. Total RNA was extracted overnight from *P. aeruginosa* bacterial cultures treated and untreated with EOs. All the details about this experimental part were previously published [32,63].

#### 3.5.6. The Synergistic Activity with Antibiotics

The antibiotic susceptibility of the *S. aureus* and *P. aeruginosa* strains was tested by the disk-diffusion method (Kirby–Bauer), according to the CLSI recommendations. The standardized bacterial suspensions were seeded onto a solid medium (Mueller–Hinton agar), as described for the qualitative screening. Two replicate plates were prepared for each strain [72]. For establishing the EOs’ influence on the antibiotic susceptibility, 10 μL of each EO stock solution (essential oil: DMSO 1:1, *v/v*) were placed on each antibiotic disk, with one replicate per strain. Plates were incubated for 16–18 h at 35 ± 2 °C. The results were read by measuring the diameter of the inhibition zones by using a hand-held caliper with a ruler, as generated by the different antibiotics comparatively to the antibiotics–EOs solution combination.

### 3.6. The Soluble Enzymatic Virulence Factors

The microbial strains were cultivated in liquid medium (nutrient broth) with and without the addition of subinhibitory concentrations of the tested EOs stock solution. The obtained overnight bacterial cultures were spotted onto special media for assessing the following virulence factors production [70,73].

Plate haemolysis: the strains were streaked on blood Sabouraud agar plates containing 5% (*v*/*v*) sheep blood in order to obtain isolated colonies. After incubation at 37 °C for 24 h the clear zone (total lysis of the red blood cells) around the colonies was registered as positive reaction.

Gelatinase activity: determined by using 3% gelatine agar as substrate medium. After incubation at 37 °C up to 48 h, a clear zone surrounding the growth area indicated gelatine proteolysis (gelatinase presence).

Caseinase activity: determined using 15% soluble casein agar as substrate. The strains were spotted and after incubation at 37 °C for 24 h, a precipitation zone surrounding the bacteria growth indicated the casein production.

DNA-se production: studied using DNA agar medium. The strains were spotted and after incubation at 37 °C for 24 h, a drop of HCl 1N solution was added upon the spotted cultures; a clearing zone around the culture was interpreted as positive reaction.

Lipase production: the cultures were spotted on Tween 80 agar with a substrate at a final concentration of 1% and were incubated at 37 °C up to 7 days. An opaque (precipitation) zone around the spot was registered as positive reaction.

Lecithinase production: the cultures were spotted into 2.5% yolk agar and were incubated at 37 °C for 7 days. An opaque (precipitation) zone around the spot indicated the lecithinase production.

### 3.7. Statistical Analysis

All experiments were done in triplicate, but the MIC and MBEC had the same values, so they were not expressed as the mean ± SD. The results obtained were represented by the last concentration at which no microbial growth was observed. The statistical impact of the EOs on microbial type (Gram-positive and Gram-negative) highlighted whether the antimicrobial effect is significant on the microorganism classes. The statistical analysis was performed using GraphPad Prism v9 (paired t-test). A *p* value < 0.05 was considered statistically significant. Significance values for antimicrobial activity against Gram-positive and Gram-negative strains are shown as *** *p* < 0.001.

## 4. Conclusions

Our study revealed that the EOs extracted from the coniferous species *L. decidua*, *P. nigra*, *P. abies* and *P. menziesii* exhibited significant antimicrobial features, in many cases equal or superior to those obtained for the major compounds, demonstrating the synergistic effect of the active compounds found in the EOs. They inhibited the microbial growth of a large number of reference and clinical and resistant *P. aeruginosa* and *S. aureus* strains as well as of the reference *C. albicans* strain, with MIC values varying from 6.25 to 100 µL/mL and the most susceptible strains being the Gram-positive and fungal ones. The most intensive and broad-spectrum microbicidal effect was exhibited by the *P. nigra* EOs. A synergistic effect with some antibiotics recommended to be tested against *S. aureus* strains (i.e., oxacillin, tetracycline, erythromycin and gentamycin) was also observed.

The subinhibitory concentrations of the tested EOs inhibited the adherence and the expression of the soluble virulence factors and modulated the QS genes’ expression in *P. aeruginosa*. All these features make the tested EOs promising leads for the development of novel antimicrobial strategies.

## Figures and Tables

**Table 1:** The chemical composition of *P. abies*, *L. decidua*, *P. menziesii* and *P. nigra* EOs obtained by gas chromatography coupled with mass spectrometry.

No.	Compound Name	RI Exp	RI ^a^ Lit	Relative Area (%)			
				Pa ^b^	Ld ^b^	Pm ^b^	Pn ^b^
1	4-hexen-1-ol	872	879	Tr	0.42 ± 0.03	0.10 ± 0.01	
2	santene	887	888	3.83 ± 1.10			
3	tricyclene	921	926	1.23 ± 0.23	0.10 ± 0.01	0.09 ± 0.03	0.11 ± 0.01
4	α-thujene	928	931		Tr	0.27 ± 0.01	0.08 ± 0.04
5	α-pinene	934	939	11.64 ± 1.34	26.99 ± 2.57	18.42 ± 2.29	74.27 ± 2.73
6	camphene	950	952	10.70 ± 0.25	0.52 ± 0.15	0.62 ± 0.10	1.24 ± 0.12
7	sabinene	973	973		0.15 ± 0.07	1.67 ± 0.59	0.02 ± 0.01
8	β-pinene	976	980	4.62 ± 1.34	8.20 ± 0.83	49.84 ± 3.57	4.33 ± 0.55
9	β-myrcene	991	991	2.26 ± 0.49	2.05 ± 0.52	1.17 ± 0.12	0.70 ± 0.18
10	α-phellandrene	1003	1005	0.13 ± 0.07	0.32 ± 0.18	0.15 ± 0.01	0.05 ± 0.01
11	δ-3-carene	1006	1009	0.89 ± 0.21	5.97 ± 1.19	0.66 ± 0.10	Tr
12	α-terpinene	1016	1017	0.08 ± 0.03	0.38 ± 0.10	0.87 ± 0.19	0.05 ± 0.01
13	p-cymene	1024	1026	0.05 ± 0.01	0.06 ± 0.01	0.36 ± 0.14	0.04 ± 0.01
14	limonene	1029	1031	21.14 ± 2.27	6.69 ± 0.93	3.58 ± 0.26	7.06 ± 0.78
15	1,8-cineole	1032	1033	0.18 ± 0.11	0.22 ± 0.01		
16	(Z)-β-ocimene	1040	1040	Tr		Tr	Tr
17	(E)-β-ocimene	1050	1050	0.05 ± 0.01			0.50 ± 0.21
18	γ-terpinene	1060	1062	0.07 ± 0.02	0.48 ± 0.23	1.38 ± 0.31	0.08 ± 0.06
19	α-terpinolene	1085	1084	0.64 ± 0.23	1.59 ± 0.54	3.71 ± 1.02	0.22 ± 0.07
20	linalool	1100	1100	Tr	0.07 ± 0.04		0.04 ± 0.03
21	fenchol	1117	1117	0.07 ± 0.01	0.14 ± 0.10		
22	α-campholenal	1126	1027	0.11 ± 0.03			0.10 ± 0.00
23	camphor	1145	1145	0.06 ± 0.03			
24	ethyl benzoate	1169	1170		0.49 ± 0.01		
25	β-terpineol	1154	1159	0.07 ± 0.02			
26	borneol	1171	1171	0.78 ± 0.06		0.09 ± 0.01	0.12 ± 0.06
27	terpinen-4-ol	1180	1179	0.08 ± 0.00	0.60 ± 0.33	2.24 ± 0.18	0.04 ± 0.01
28	α-terpineol	1193	1197	0.57 ± 0.04	2.27 ± 0.62	1.86 ± 0.44	0.35 ± 0.14
29	estragole	1196	1195	0.27 ± 0.01			
30	fenchyl acetate	1216	1220	0.05 ± 0.01			
31	methyl thymyl ether	1231	1235		Tr	Tr	0.04 ± 0.01
32	bornyl acetate	1283	1285	11.08 ± 1.80	1.26 ± 0.04	0.20 ± 0.04	1.21 ± 0.17
33	sabinyl acetate	1293	1293	0.16 ± 0.01			
34	δ-elemene	1334	1337			0.6 ± 0.43	
35	terpenyl acetate	1347	1351		0.18 ± 0.01		
36	α-longipinene	1349	1351	0.34 ± 0.03			
37	citronellyl acetate	1351	1354	0.04 ± 0.01	0.06 ± 0.01	Tr	
38	longicyclene	1371	1373	0.05 ± 0.03			
39	α-copaene	1375	1376	0.06 ± 0.03	0.08 ± 0.03		0.03 ± 0.03
40	geranyl acetate	1380	1382	0.04 ± 0.03			0.12 ± 0.01
41	β-elemene	1387	1391	0.15 ± 0.03	0.27 ± 0.03	0.42 ± 0.26	
42	longifolene	1404	1408	0.50 ± 0.09			
43	trans-caryophyllene	1416	1415	1.17 ± 0.13	2.68 ± 0.18	0.34 ± 0.16	1.99 ± 0.30
44	γ-elemene	1428	1430			0.13 ± 0.01	
45	trans-α-bergamotene	1432	1436	0.04 ± 0.01		0.48 ± 0.04	
46	α-humulene	1453	1452	1.23 ± 0.04	1.12 ± 0.13	1.07 ± 0.54	0.27 ± 0.03
47	ethyl cinnamate	1464	1460		0.11 ± 0.01		
48	β-cadinene	1470	1472	0.15 ± 0.06	0.16 ± 0.07		
49	γ-muurolene	1473	1477	0.24 ± 0.03	0.76 ± 0.06	0.20 ± 0.04	0.16 ± 0.04
50	germacrene D	1478	1480	0.76 ± 0.07	19.80 ± 4.40	5.47 ± 2.70	2.74 ± 1.07
51	ledene	1485	1487		0.08 ± 0.01		
52	phenylethyl isovalerate	1489	1489				0.03 ± 0.01
53	valencene	1491	1490	0.10 ± 0.01			0.06 ± 0.04
54	β-selinene	1485	1485	0.05 ± 0.04		0.38 ± 0.18	
55	α-selinene	1493	1494	0.19 ± 0.08			
56	α-muurolene	1496	1499	0.75 ± 0.13	1.01 ± 0.18	0.09 ± 0.01	0.13 ± 0.06
57	α-farnesene	1504	1508	0.25 ± 0.01			
58	γ-cadinene	1510	1514	0.86 ± 0.31	0.72 ± 0.04	0.20 ± 0.01	0.11 ± 0.03
59	δ-cadinene	1517	1523	4.21 ± 0.69	4.52 ± 0.66	0.74 ± 0.01	0.34 ± 0.06
60	zonarene	1521	1526	0.15 ± 0.07	0.16 ± 0.06		
61	cadina-1,4-diene	1531	1532	0.07 ± 0.04	0.10 ± 0.00		
62	α-cadinene	1536	1538	0.14 ± 0.07	0.17 ± 0.04		
63	trans-α-bisabolene	1541	1544			0.95 ± 0.24	
64	germacrene B	1553	1560		0.10 ± 0.04	0.30 ± 0.00	
65	nerolidol	1560	1565	0.06 ± 0.00	0.04 ± 0.01		
66	γ-eudesmol	1631	1630			0.11 ± 0.01	
67	α-muurolol	1643	1645	2.15 ± 0.28	2.55 ± 0.54		
68	δ-cadinol	1646	1646	0.32 ± 0.05			
69	α-cadinol	1655	1656	3.78 ± 0.76	4.11 ± 0.88	0.36 ± 0.21	
70	manool	2053	2056	9.40 ± 1.85			
71	verticillol	2102	2106 ^c^				2.14 ± 1.60
TOTAL				98.06 ± 0.97	97.75 ± 2.23	99.13 ± 0.04	98.77 ± 1.68
Monoterpene hydrocarbons				57.77 ± 3.85	52.90 ± 0.53	82.50 ± 6.68	88.56 ± 4.83
Sesquiterpene hydrocarbons				11.43 ± 1.78	31.63 ± 6.53	11.22 ± 4.35	5.83 ± 2.09
Monoterpene alcohols and esters				12.90 ± 2.97	4.01 ± 1.85	4.39 ± 0.31	1.86 ± 0.32
Sesquiterpene alcohols				6.28 ± 0.48	6.69 ± 1.43	0.39 ± 0.31	0.00
Diterpene alcohols				9.40 ± 1.85	0	0	2.14 ± 1.60

^a^ RI, the retention index relative to C_8_–C_24_ n-alkanes on a DB-5MS column. ^b^ Pa: *P. abies*; Ld: *L. decidua*; Pm: *P. menziesii*; Pn: *P. nigra*. ^c^ [33].

**Table 2:** The MIC (μL/mL) a and MBEC (μL/mL) b values for *P. abies*, *L. decidua*, *P. menziesii* and *P. nigra* essential oils and for some pure compounds against Gram-positive bacteria, Gram-negative bacteria and *C. albicans*.

Strain	a	Pin	Lim	Phel	Bor	Cam	Cin	Ner	*Pa*	*Ld*	*Pm*	*Pn*	Gen
	b												
*S. aureus* ATCC 25923	a	50	25	50	50	50	25	50	25	12.5	6.25	12.5	0.48
	b	25	12.5	25	25	25	12.5	25	12.5	6.25	3.13	6.25	n.t.
*S. aureus* 19 F	a	50	50	50	50	50	50	50	6.25	50	25	25	0.48
	b	25	25	25	25	25	25	25	3.13	25	12.5	12.5	n.t.
*S. aureus* 8 V	a	25	50	50	50	50	50	50	20	25	25	25	0.96
	b	12.5	25	25	25	25	25	25	10	12.5	12.5	12.5	n.t.
*S. aureus* 12 H	a	12.5	50	50	50	50	50	50	6.25	50	25	6.25	0.96
	b	6.25	25	25	25	25	25	25	3.13	25	12.5	3.13	n.t.
*S. aureus* 35 PL	a	12.5	50	50	50	50	50	50	6.25	25	25	6.25	2.88
	b	6.25	25	25	25	25	25	25	3.13	12.5	12.5	3.13	n.t.
*Pseudomonas aeruginosa* ATCC 27853	a	25	50	50	50	50	50	50	50	50	25	25	1.96
	b	12.5	25	25	25	25	25	25	25	25	2512.5	12.5	n.t.
*P. aeruginosa* 1 H	a	25	25	50	50	50	50	50	50	50	25	25	7.84
	b	12.5	12.5	25	25	25	25	25	25	25	12.5	12.5	n.t.
*P. aeruginosa* 61/2	a	50	50	50	50	50	50	50	50	50	25	25	15.68
	b	25	25	25	25	25	25	25	25	25	12.5	12.5	n.t.
*P. aeruginosa* 399	a	50	50	25	25	25	25	25	50	50	50	25	15.68
	b	25	25	12.5	12.5	12.5	12.5	12.5	25	25	25	12.5	n.t.
*P. aeruginosa* 261/1	a	50	50	25	25	25	25	25	50	50	50	25	7.84
	b	25	25	12.5	12.5	12.5	12.5	12.5	25	25	25	12.5	n.t.
*B. subtilis* 6633	a	n.t.	n.t.	n.t.	n.t.	n.t.	n.t.	n.t.	6.25	50	25	6.25	0.24
	b	n.t.	n.t.	n.t.	n.t.	n.t.	n.t.	n.t.	3.13	25	12.5	3.13	n.t.
*E. faecalis* ATCC 29212	a	n.t.	n.t.	n.t.	n.t.	n.t.	n.t.	n.t.	25	50	50	25	7.68
	b	n.t.	n.t.	n.t.	n.t.	n.t.	n.t.	n.t.	12.5	25	25	12.5	n.t.
*E. coli* ATCC 25922	a	n.t.	n.t.	n.t.	n.t.	n.t.	n.t.	n.t.	50	50	50	25	0.48
	b	n.t.	n.t.	n.t.	n.t.	n.t.	n.t.	n.t.	25	25	25	12.5	n.t.
*C. albicans* ATCC 10231	a	n.t.	n.t.	n.t.	n.t.	n.t.	n.t.	n.t.	6.25	25	25	6.25	n.t.
	b	n.t.	n.t.	n.t.	n.t.	n.t.	n.t.	n.t.	3.13	12.5	12.5	3.13	n.t.

a: MIC (μL/mL): minimum inhibitory concentration; b: MBEC (μL/mL): minimum biofilm eradication concentration on an inert substrate; Pin: α-pinene; Lim: (+)-limonene; Phel: phellandrene; Bor: borneol; Cam: camphor; Cin: 1,8-cineole; Ner: nerolidol; Pa: *P. abies*; Ld: *L. decidua*; Pm: *P. menziesii*; Pn: *P. nigra*, Gen: gentamycin (μg/mL), n.t. = not tested.

**Table 3:** The synergistic activity of the tested EOs with different antibiotics.

Strains	Sample	The Diameter of the Inhibition Zones (mm)						
		oxa	cli	cip	tet	gen	pen	ery
*S. aureus* ATCC 25923	control	18	32	22	24	22	26	26
	*P. abies*	18	40	26	23	23	26	30
	*L. decidua*	18	30	24	22	22	26	22
	*P.* *menziesii*	18	26	24	23	21	26	26
	*P. nigra*	18	38	27	22	24	26	23
*S. aureus* 19 F	control	10	32	28	22	19	0	22
	*P. abies*	10	28	33	21	21	8	22
	*L. decidua*	10	40	32	20	25	13	27
	*P.* *menziesii*	10	36	28	22	23	18	25
	*P. nigra*	10	34	29	22	23	7	27
*S. aureus* 8 V	control	11	38	25	20	19	0	10
	*P. abies*	11	34	27	21	21	0	9
	*L. decidua*	11	33	27	21	21	0	11
	*P.* *menziesii*	11	32	27	21	21	0	11
	*P. nigra*	11	36	27	21	21	0	12
S. *aureus* 12 H	control	10	34	24	20	20	15	11
	*P. abies*	10	36	27	26	21	19	12
	*L. decidua*	10	38	28	22	19	15	12
	*P.* *menziesii*	10	36	25	20	19	15	11
	*P. nigra*	10	44	30	30	23	24	24
S. *aureus* 35 PL	control	14	25	23	0	18	0	0
	*P. abies*	14	26	25	0	18	0	0
	*L. decidua*	14	26	23	0	20	0	0
	*P.* *menziesii*	14	28	24	0	19	8	0
	*P. nigra*	14	29	24	9	20	0	0

oxa = oxacillin; cli = clindamycin; cip = ciprofloxacin; tet = tetracycline; gen = gentamycin; pen = penicillin; ery = erythromycin.

**Table 4:** The number of strains in which the inhibition of at least one virulence factor expression was noticed in the presence of the EOs and of their major compounds.

	Borneol	Camfor	1,8-Cineole	Limonene	*α*-Pinene	*Pa* ^a^	*Ld* ^a^	*Pm* ^a^	*Pn* ^a^
*S. aureus*									
Total no. of strains	10	10	10	10	10	10	10	10	10
DN-ase	1	1	1	1	1	1	1	1	1
Lipase	1	1	2	1	2	1	1	1	1
Lecithinase	3	3	4	3	3	5	5	3	5
Haemolysins	8	8	9	9	9	9	9	9	9
Caseinase	3	3	3	3	4	5	3	3	3
Siderophore-like	5	5	7	7	8	7	6	5	6
*P. aeruginosa*									
Total no. of strains	10	10	10	10	10	10	10	10	10
DN-ase	10	9	10	9	10	10	10	10	9
Lipase	5	6	6	5	5	6	5	6	6
Lecithinase	4	5	5	5	6	6	6	5	5
Haemolysins	6	7	6	7	7	6	6	6	6
Caseinase	9	9	8	9	0	0	0	0	0
Siderophore-like	10	7	10	10	10	10	10	10	10

^a^ Pa: *P. abies*; Ld: *L. decidua*; Pm: *P.* *menziesii;* Pn: *P. nigra*.

## Data Availability

Data is contained within the article or supplementary material.

## Supplementary Materials

The following are available online at https://www.mdpi.com/article/10.3390/ph14111159/s1, Figure S1. Chromatogram of the Larix decidua essential oil. Figure S2. Chromatogram of the Pseudotsuga menziesii essential oil. Figure S3. Chromatogram of the Pinus nigra volatile essential oil.

## References

[1] Bakkali F., Averbeck S., Averbeck D., Idaomar M. (2008). Biological effects of essential oils—A review. Food Chem. Toxicol..

[2] Tajkarimi M.M., Ibrahim S.A., Cliver D.O. (2010). Antimicrobial herb and spice compounds in food. Food Control.

[3] Schnitzler P., Astani A., Reichling J. (2011). Screening for antiviral activities of isolated compounds from essential oils. Evid.-Based Complement. Altern. Med..

[4] Silva F., Ferreira S., Duarte A., Mendonça D.I., Domingues F.C. (2011). Antifungal activity of *Coriandrum sativum* essential oil, its mode of action against Candida species and potential synergism with amphotericin B. Phytomedicine.

[5] Schuldt T., Dommerich S., Pau H.-W., Kramp B. (2010). Die mikrobiologische Oberflächenbesiedlung verschiedener Stimmprothesenarten in der Zeitkinetik. Laryngo-Rhino-Otologie.

[6] LaSarre B., Federle M.J. (2013). Exploiting Quorum Sensing To Confuse Bacterial Pathogens. Microbiol. Mol. Biol. Rev..

[7] Milutinović M., Dimitrijević-Branković S., Rajilić-Stojanović M. (2021). Plant Extracts Rich in Polyphenols as Potent Modulators in the Growth of Probiotic and Pathogenic Intestinal Microorganisms. Front Nutr..

[8] Biradar B., Devi P. (2011). Quorum sensing in plaque biofilms: Challenges and future prospects. J. Contemp. Dent. Pract..

[9] Marinas I.C., Oprea E., Buleandra M., Badea I.A., Tihauan B.M., Marutescu L., Angheloiu M., Matei E., Chifiriuc M.C. (2021). Chemical Composition, Antipathogenic and Cytotoxic Activity of the Essential Oil Extracted from *Amorpha fruticosa* Fruits. Molecules.

[10] Bhardwaj K., Islam M.T., Jayasena V., Sharma B., Sharma S., Sharma P., Kuča K., Bhardwaj P. (2020). Review on essential oils, chemical composition, extraction, and utilization of some conifers in Northwestern Himalayas. Phyther. Res..

[11] Radulescu V., Saviuc C., Chifiriuc C., Oprea E., Ilies D.C., Marutescu L., Lazar V. (2011). Chemical Composition and Antimicrobial Activity of Essential Oil from Shoots Spruce (*Picea abies* L). Rev. Chim..

[12] Holubová V., Hrdlička P., Kubáň V. (2001). Age and space distributions of monoterpenes in fresh needles of *Picea abies* (L) Karst. determined by gas chromatography-mass spectrometry. Phytochem. Anal..

[13] Kupcinskiene E., Stikliene A., Judzentiene A. (2008). The essential oil qualitative and quantitative composition in the needles of *Pinus sylvestris* L. growing along industrial transects. Environ. Pollut..

[14] Turtola S., Sallas L., Holopainen J.K., Julkunen-Tiitto R., Kainulainen P. (2006). Long-term exposure to enhanced UV-B radiation has no significant effects on growth or secondary compounds of outdoor-grown Scots pine and Norway spruce seedlings. Environ. Pollut..

[15] Judžentienė A., Sližytė J., Stiklienė A., Kupčinskiene E. (2006). Characteristics of essential oil composition in the needles of young Scots pine (*Pinus sylvestris* L.) stands growing along an aerial ammonia gradient. Chemija.

[16] Lee J.-H. (2009). Comparison of Chemical Compositions and Antimicrobial Activities of Essential Oils from Three Conifer Trees; *Pinus densiflora*, *Cryptomeria japonica*, and *Chamaecyparis obtusa*. J. Microbiol. Biotechnol..

[17] Metsämuuronen S., Siren H. (2014). Antibacterial Compounds in Predominant Trees in Finland: Review. J. Bioprocess. Biotech..

[18] Snieškienė V., Stankevičienė A., Varkulevičienė J. (2008). The Effect of The Essential Oils on Micromycetes Isolated from Plants. Zemdirb. Agric. Kėdainių Raj. Liet. Zemdirb. Inst..

[19] Krauze-Baranowska M., Mardarowicz M., Wiwart M., Pobłocka L., Dynowska M. (2002). Antifungal Activity of the Essential Oils from Some Species of the Genus Pinus. Z. Naturforsch. C.

[20] Tesevic V., Milosavljevic S., Vajs V., Djordjevic I., Sokovic M., Lavadinovic V., Novakovic M. (2009). Chemical composition and antifungal activity of the essential oil of Douglas fir (*Pseudosuga menziesii* mirb. Franco) from Serbia. J. Serb. Chem. Soc..

[21] Politeo O. (2011). Chemical composition and evaluation of acetylcholinesterase inhibition and antioxidant activity of essential oil from Dalmatian endemic species *Pinus nigra* Arnold ssp. dalmatica (Vis.) Franco. J. Med. Plants Res..

[22] Yang X., Zhao H., Wang J., Meng Q., Zhang H., Yao L., Zhang Y., Dong A., Ma Y., Wang Z. (2010). Chemical Composition And Antioxidant Activity Of Essential Oil Of Pine Cones Of Pinus Armandii From The Southwest Region of China. J. Med. Plants Res..

[23] Kılıç Ö., Kocak A. (2014). Essential oil composition of six *Pinus* L. taxa (*Pinaceae*) from Canada and their chemotaxonomy. J. Agric. Sci. Technol..

[24] Nikolić B., Ristić M., Bojović S., Marin P.D. (2007). Variability of the Needle Essential Oils of *Pinus heldreichii* from Different Populations in Montenegro and Serbia. Chem. Biodivers..

[25] Dob T., Berramdane T., Dahmane D., Chelghoum C. (2005). Chemical Composition of the Needles Oil of *Pinus canariensis* from Algeria. Chem. Nat. Compd..

[26] Almaarri K., Alamir L., Junaid Y., Xie D.-Y. (2010). Volatile compounds from leaf extracts of *Juniperus excelsa* growing in Syria via gas chromatography mass spectrometry. Anal. Methods.

[27] Tumen I., Hafizoglu H., Kilic A., Dönmez I.E., Sivrikaya H., Reunanen M. (2010). Yields and Constituents of Essential Oil from Cones of *Pinaceae* spp. Natively Grown in Turkey. Molecules.

[28] Dayisoylu K.S., Alma M.H. (2009). Chemical analysis of essential oils from cone’s rosin of Cilician fir (*Abies cilicica* subsp. cilicica). Afr. J. Biotechnol..

[29] Duquesnoy E., Castola V., Casanova J. (2007). Composition and chemical variability of the twig oil of *Abies alba* Miller from Corsica. Flavour Fragr. J..

[30] Amri I., Hanana M., Jamoussi B., Hamrouni L. (2017). Essential oils of Pinus nigra J.F. Arnold subsp. laricio Maire: Chemical composition and study of their herbicidal potential. Arab. J. Chem..

[31] Rădulescu V., Ilies D.C., Voiculescu I., Biris I.A., Craciunescu A. (2013). Determination of ascorbic acid in shoots from different coniferous species by HPLC. Farmacia.

[32] Saviuc C., Cotar A.I., Holban A.M., Banu O., Grumezescu A.M., Carmen Chifiriuc M. (2013). Phenotypic and molecular evaluation of *Pseudomonas aeruginosa* and *Staphylococcus aureus* virulence patterns in the presence of some essential oils and their major compounds. Lett. Appl. NanoBioSci.

[33] Awadh Ali N.A., Wurster M., Arnold N., Teichert A., Schmidt J., Lindequist U., Wessjohan L. (2008). Chemical Composition and Biological Activities of Essential Oils from the Oleogum Resins of Three Endemic Soqotraen *Boswellia* Species. Rec. Nat. Prod..

[34] Garcia G., Garcia A., Gibernau M., Bighelli A., Tomi F. (2017). Chemical compositions of essential oils of five introduced conifers in Corsica. Nat. Prod. Res..

[35] Mofikoya O.O., Mäkinen M., Jänis J. (2020). Chemical Fingerprinting of Conifer Needle Essential Oils and Solvent Extracts by Ultrahigh-Resolution Fourier Transform Ion Cyclotron Resonance Mass Spectrometry. ACS Omega.

[36] Runyoro D., Ngassapa O., Vagionas K., Aligiannis N., Graikou K., Chinou I. (2010). Chemical composition and antimicrobial activity of the essential oils of four *Ocimum* species growing in Tanzania. Food Chem..

[37] Andrews R.E., Parks L.W., Spence K.D. (1980). Some effects of Douglas fir terpenes on certain microorganisms. Appl. Environ. Microbiol..

[38] Uribe S., Ramirez J., Pena A. (1985). on Yeast Membrane Functions. Yeast.

[39] Helander I.M., Alakomi H.L., Latva-Kala K., Mattila-Sandholm T., Pol I., Smid E.J., Gorris L.G.M., Von Wright A. (1998). Characterization of the Action of Selected Essential Oil Components on Gram-Negative Bacteria. J. Agric. Food Chem..

[40] Magwa M.L., Gundidza M., Gweru N., Humphrey G. (2006). Chemical composition and biological activities of essential oil from the leaves of *Sesuvium portulacastrum*. J. Ethnopharmacol..

[41] Filipowicz N., Kaminski M., Kurlenda J., Asztemborska M., Ochocka J.R. (2003). Antibacterial and antifungal activity of juniper berry oil and its selected components. Phyther. Res..

[42] Chifiriuc M.C., Banu O., Bleotu C., Lazǎr V. (2011). Interaction of bacteria isolated from clinical biofilms with cardiovascular prosthetic devices and eukaryotic cells. Anaerobe.

[43] Lazar V., Holban A.M., Curutiu C., Chifiriuc M.C. (2021). Modulation of Quorum Sensing and Biofilms in Less Investigated Gram-Negative ESKAPE Pathogens. Front. Microbiol..

[44] Bilcu M., Grumezescu A.M., Oprea A.E., Popescu R.C., Mogoșanu G.D., Hristu R., Stanciu G.A., Mihailescu D.F., Lazar V., Bezirtzoglou E. (2014). Efficiency of Vanilla, Patchouli and Ylang Ylang Essential Oils Stabilized by Iron Oxide@C14 Nanostructures against Bacterial Adherence and Biofilms Formed by *Staphylococcus aureus* and *Klebsiella pneumoniae* Clinical Strains. Molecules.

[45] Ciubuca B.-M., Saviuc C.-M., Chifiriuc M.-C., Lazar V. (2016). Microbial Resistance to Natural Compounds: Challenges for Developing Novel Alternatives to Antibiotics. Curr. Org. Chem..

[46] Kovač J., Šimunović K., Wu Z., Klančnik A., Bucar F., Zhang Q., Možina S.S. (2015). Antibiotic Resistance Modulation and Modes of Action of (-)-α-Pinene in Campylobacter jejuni. PLoS ONE.

[47] Ayaz M., Ullah F., Sadiq A., Ullah F., Ovais M., Ahmed J., Devkota H.P. (2019). Synergistic interactions of phytochemicals with antimicrobial agents: Potential strategy to counteract drug resistance. Chem. Biol. Interact..

[48] Eleraky N.E., Allam A., Hassan S.B., Omar M.M. (2020). Nanomedicine Fight against Antibacterial Resistance: An Overview of the Recent Pharmaceutical Innovations. Pharmaceutics.

[49] Miladinović D.L., Ilić B.S., Kocić B.D., Marković M.S., Miladinović L.C. (2016). In Vitro Trials of *Dittrichia graveolens* Essential Oil Combined with Antibiotics. Nat. Prod. Commun..

[50] Sobieszczańska N., Myszka K., Szwengiel A., Majcher M., Grygier A., Wolko Ł. (2020). Tarragon essential oil as a source of bioactive compounds with anti-quorum sensing and anti-proteolytic activity against *Pseudomonas* spp. isolated from fish—In vitro, in silico and in situ approaches. Int. J. Food Microbiol..

[51] Lu L., Li M., Yi G., Liao L., Cheng Q., Zhu J., Zhang B., Wang Y., Chen Y., Zeng M. (2021). Screening Strategies for Quorum Sensing Inhibitors in Combating Bacterial Infection. J. Pharm. Anal..

[52] Luciardi M.C., Blázquez M.A., Alberto M.R., Cartagena E., Arena M.E. (2021). Lemon oils attenuate the pathogenicity of pseudomonas aeruginosa by quorum sensing inhibition. Molecules.

[53] Baban Zadeh P., Khaledi A., Esmaeili D. (2019). *Satureja khuzestanica* essential oil against quorum sensing of pseudomonas aeroginosa using RT-PCR. Iran. J. Public Health.

[54] Li W.R., Ma Y.K., Xie X.B., Shi Q.S., Wen X., Sun T.L., Peng H. (2019). Diallyl disulfide from garlic oil inhibits *Pseudomonas aeruginosa* quorum sensing systems and corresponding virulence factors. Front. Microbiol..

[55] Lou Z., Letsididi K.S., Yu F., Pei Z., Wang H., Letsididi A.R. (2019). Inhibitive effect of eugenol and its nanoemulsion on quorum sensing–mediated virulence factors and biofilm formation by *Pseudomonas aeruginosa*. J. Food Prot..

[56] Kumar L., Chhibber S., Kumar R., Kumar M., Harjai K. (2015). Zingerone silences quorum sensing and attenuates virulence of *Pseudomonas aeruginosa*. Fitoterapia.

[57] Kostylev M., Kim D.Y., Smalley N.E., Salukhe I., Peter Greenberg E., Dandekar A.A. (2019). Evolution of the *Pseudomonas aeruginosa* quorum-sensing hierarchy. Proc. Natl. Acad. Sci. USA.

[58] Jack A.A., Khan S., Powell L.C., Pritchard M.F., Beck K., Sadh H., Sutton L., Cavaliere A., Florance H., Rye P.D. (2018). Alginate Oligosaccharide-Induced Modification of the lasI-lasR and rhlI-rhlR Quorum-Sensing Systems in *Pseudomonas aeruginosa*. Antimicrob. Agents Chemother..

[59] Dickey S., Cheung G., Otto M. (2017). Different drugs for bad bugs: Antivirulence strategies in the age of antibiotic resistance. Nat. Rev. Drug Discov..

[60] Allen R.C., Popat R., Diggle S.P., Brown S.P. (2014). Targeting virulence: Can we make evolution-proof drugs?. Nat. Rev. Microbiol..

[61] Rasko D.A., Sperandio V. (2010). Anti-virulence strategies to combat bacteria-mediated disease. Nat. Rev. Drug Discov..

[62] Sintchenko V., Timms V., Sim E., Rockett R., Bachmann N., O’Sullivan M., Marais B. (2021). Microbial Genomics as a Catalyst for Targeted Antivirulence Therapeutics. Front. Med..

[63] Cotar A.I., Chifiriuc M.C., Dinu S., Pelinescu D., Banu O., Lazăr V. (2010). Quantitative real-time PCR study of the influence of probiotic culture soluble fraction on the expression of *Pseudomonas aeruginosa* quorum sensing genes. Roum. Arch. Microbiol. Immunol..

[64] Neguţ A.C., Chifiriuc M.C., Săndulescu O., Streinu-Cercel A., Oprea M., Drăgulescu E.C., Gheorghe I., Berciu I., Coralia B., Popa M. (2016). Bacteriophage-driven inhibition of biofilm formation in Staphylococcus strains from patients attending a Romanian reference center for infectious diseases. FEMS Microbiol. Lett..

[65] Marinas I.C., Oprea E., Chifiriuc M.C., Badea I.A., Buleandra M., Lazar V. (2015). Chemical Composition and Antipathogenic Activity of *Artemisia annua* Essential Oil from Romania. Chem. Biodivers..

[66] Commission National Medicines Agency (1993). Romanian Pharmacopoeia.

[67] Adams R.P. (1996). Identification of Essential Oils by Ion Trap Mass Spectroscopy.

[68] Acree T.E., Arn H. Flavornet and Human Odor Space. www.flavornet.org.

[69] El-Saed A.M. (2019). The Pherobase: Datebase of Pheromones and Semiochemicals. www.pherobase.org.

[70] Lazar V., Balotescu M.C., Moldovan L., Vasilescu G., Petrache L.M., Bulai D., Cernat R.C. (2005). Comparative evaluation of qualitative and quantitative methods used in the study of antifungal and antibacterial activity of hydroalcoholic vegetal extracts. Rom. Biotechnol. Lett..

[71] Norouzi F., Mansouri S., Moradi M. (2010). Comparison of cell surface hydrophobicity and biofilm formation among ESBL-and nonESBL-producing *Pseudomonas aeruginosa* clinical isolates. Afr. J. Microbiol. Res..

[72] Grădinaru A.C., Trifan A., Şpac A., Brebu M., Miron A., Aprotosoaie A.C. (2018). Antibacterial activity of traditional spices against lower respiratory tract pathogens: Combinatorial effects of *Trachyspermum ammi* essential oil with conventional antibiotics. Lett. Appl. Microbiol..

[73] Georgescu M., Gheorghe I., Curutiu C., Lazar V., Bleotu C., Chifiriuc M.C. (2016). Virulence and resistance features of *Pseudomonas aeruginosa* strains isolated from chronic leg ulcers. BMC Infect. Dis..

