# Supplementary material for: Original Contributions to the Chemical Composition, Microbicidal, Virulence-Arresting and Antibiotic-Enhancing Activity of Essential Oils from Four Coniferous Species
Source: Pharmaceuticals (Basel). 2021 Nov 13;14(11):1159. doi: 10.3390/ph14111159 (PMC8617773; doi:10.3390/ph14111159)
Supplement: Supplementary file 1 [file pharmaceuticals-14-01159-s001.zip › pharmaceuticals-1421253-supplementary.pdf]

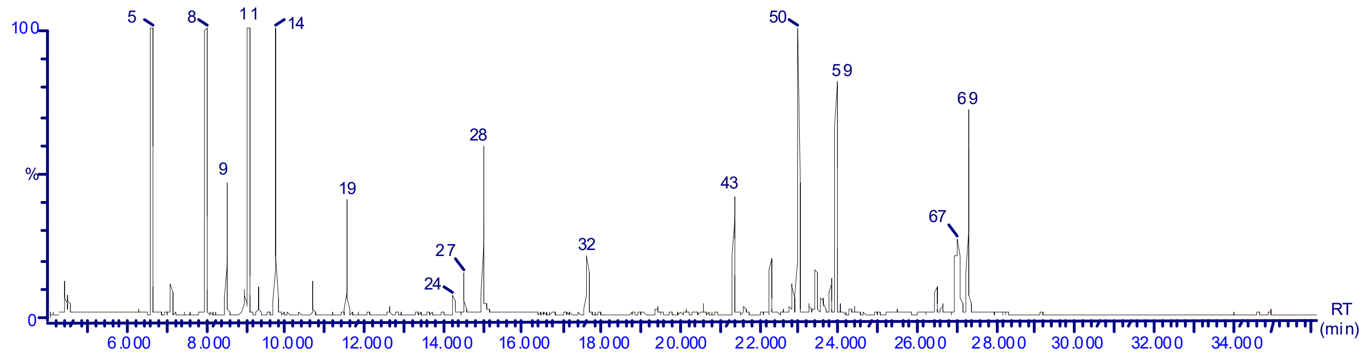

Figure S1. Chromatogram of the *Larix decidua* essential oil.

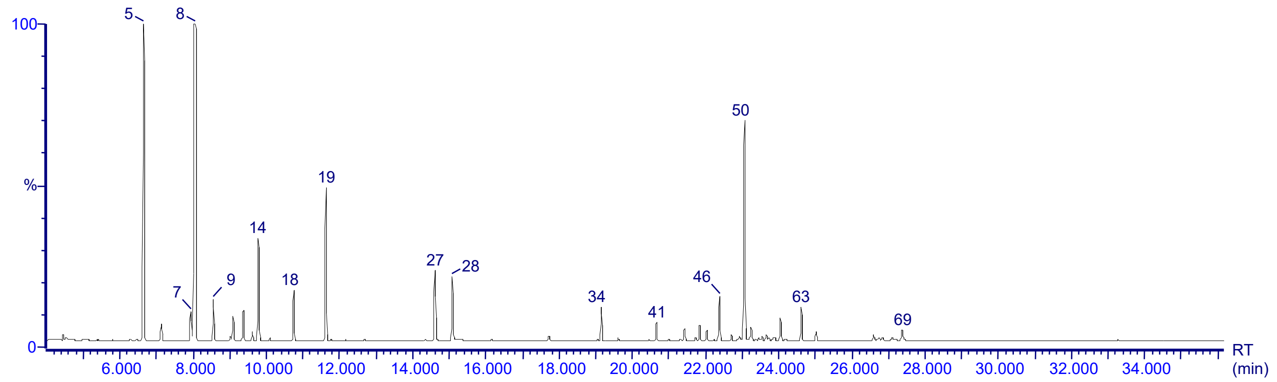

Figure S2. Chromatogram of the *Pseudotsuga menziesii* essential oil.

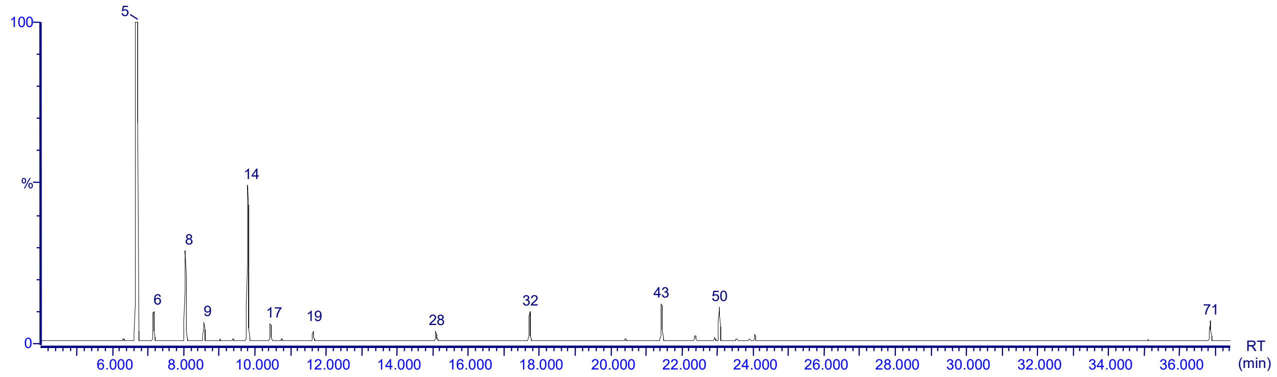

Figure S3. Chromatogram of the *Pinus nigra* volatile essential oil.
